# Fifty Years of the Double Helix

**DOI:** 10.1002/cfg.286

**Published:** 2003-04

**Authors:** Steve Oliver


					Comparative and Functional Genomics
Comp Funct Genom 2003; 4: 170.
Published online 1 April 2003 in Wiley InterScience (www.interscience.wiley.com). DOI: 10.1002/cfg.286
Editorial
Fifty years of the double helix
This April it will be 50 years since the discov-
ery of the double helical structure of DNA by
Jim Watson and Francis Crick. Since then, the
genomes of a wide range of bacteria, archaea,
viruses and several eukaryotes have been entirely
decoded, and the human genome is fast approach-
ing completion.
This issue illustrates the diversity of the field of
genomics, which was born out of their discovery.
It highlights work on a range of bacteria, plants
and animals, using a host of technologies that have
arisen since the discovery of the structure of DNA.
Comparative and Functional Genomics would
like to join with the biological research community
in congratulating Watson and Crick, once more, on
their crucial discovery and in celebrating the great
strides that have been made in our understanding
of genes and genomes since then.
Steve Oliver
Copyright  2003 John Wiley & Sons, Ltd.

				

